# Selenium-Functionalized Corn Starch as a Biodegradable GPx Mimic with High Catalytic Performance

**DOI:** 10.3390/polym13244309

**Published:** 2021-12-09

**Authors:** Shufei Jiao, Zijie Liu, Min Liu, Yongxian Liu, Shuming Zhong, Feng Wang, Yanzhen Yin

**Affiliations:** 1 School Petroleum and Chemical Engineering, Beibu Gulf University, Qinzhou 535011, China; jiaoshufei2013@163.com (S.J.); fengye83818@163.com (Z.L.); minminliu1025@163.com (M.L.); zsm_7104@163.com (S.Z.); 2 Agricultural Resource and Environment Research Institute, Guangxi Academy of Agricultural Sciences, Nanning 535007, China; liuyx@163.com

**Keywords:** OSA starch, selenium functionalization, GPxmimic, catalytic mechanism, cytotoxicity

## Abstract

Selenium-functionalized starch (Se-starch80) is one of the main functional foods used for selenium supplementation. In traditional agriculture, Se-starch has some deficiencies such as long growth cycle and unstable selenium content that prevent its antioxidant performance. In this study, Se-starch was prepared by the nucleophilic addition between NaSeH and carbon-carbon double bond of octenyl succinic anhydride waxy corn starch ester (OSA starch). Some techniques such as ^1^HNMR, XPS, SEM-EDS, XRD and FT-IR were used to characterize the relevant samples and the results showed that the modification did not destroy the starch framework significantly and the catalytic center (negative divalent selenium) was anchored on the starch framework. The intensive distribution of catalytic center on the starch surface and the hydrophobic microenvironments derived from the OSA chains furnished the Se-starch80 with a high GPx-like catalytic activity (initial reaction rate = 3.64 μM/min). This value was about 1.5 × 10^5^ times higher than that of a typical small-molecule GPx mimic (PhSeSePh). In addition, the Se-starch80, without any cytotoxicity, showed a saturated kinetic catalytic behavior that is similar to a typical enzyme. This work exemplifies a biodegradable selenium-functionalized polymer platform for the high-performing GPx mimic.

## 1. Introduction

Selenium (Se) is an important trace element that participates in the synthesis of 25 Se-containing proteins in the human body. Glutathione peroxidase (GPx), with the –SeH catalytic center, is a particularly important antioxidant enzyme that scavenges the excess free radicals in human body. GPx and therefore protects human against oxidative damage [1], inflammation [2], cancer [3], and Keshan disease [4,5]. As the continuous increase in health awareness and healthcare, it is widely concerned to obtain selenium-functionalized supplementation for maintaining the health [6,7,8]. In the traditional cultivation, Se-functionalized nutrients are mainly in the form of selenide protein deriving from wheat [9,10,11], rice [12,13,14], potato [15,16], etc. Due to the low protein content in these crops and the complex and long growth period of plant, the selenium content is unstable in targeted products. To solve this problem, in the past decade, many GPx mimics such as small molecules [17,18], block copolymers [19,20], supramolecular materials [21], and nanomaterials [22] with stable selenium component have been designed and evaluated the antioxidant efficacy. However, most of these GPx mimics is limited in the application of food and drugs since their biotoxicity and poor biodegradability. Therefore, it is still a challenge to obtain biodegradable Se-functionalized supplements with stable selenium content.

Starch, one of the most important foods for human diet, is expected to be an ideal food source of selenium supplements. However, due to the limited metabolic process, the selenium-containing starch from natural plants is rarely found so far. Compared with protein, starch reveals a more stable structure in the chemical modifications. Sun et al. [23] synthesized a novel biodegradable starch-based hydrogel by using starch and Se-containing cross-linker. Such hydrogel revealed multi stimuli responsiveness property like enzyme hydrolysis and redox responsive cleavage, making it a promising biomedical candidate of controlled drug delivery. Kazemi et al. [24] prepared colloidal selenium nanoparticles in a starch matrix and showed its nontoxicity in colorectal cancer SW480 cell via the MTT assay. Moreover, the selenium nanoparticles encapsulated in a starch matrix (NC Se/St), as an effective and environmentally safe agent, revealed a targeted low-dose delivery to bacterial phytopathogens [25]. These studies indicated that the starch combined with selenium is biodegradable and safe in the medical application. Unfortunately, the Se-containing starch with GPx-like catalytic activity has not been synthesized and studied. 

**Scheme 1:**
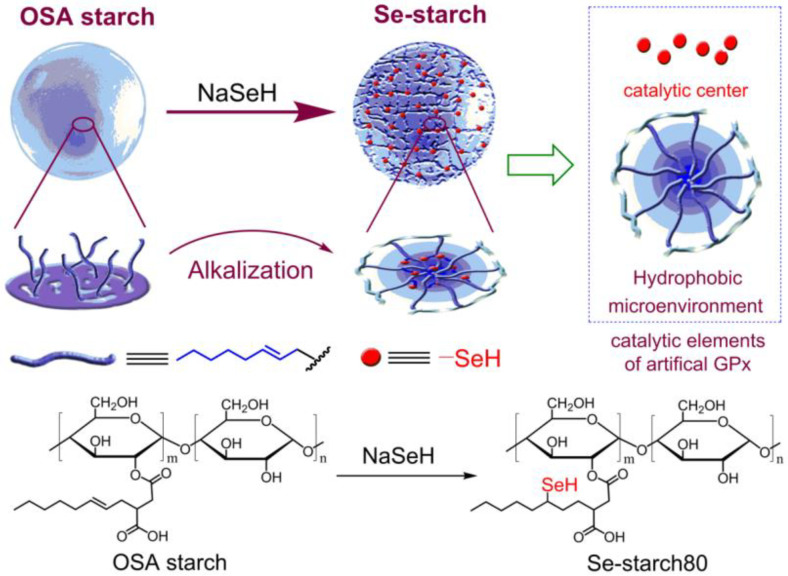
The schematic illustration for preparing selenium-functionalized waxy corn starch.

It is well-known that the octenyl succinic anhydride starch ester (OSA starch) is a typical modified starch with wide applications in foods and medicines [26,27]. In the OSA starch skeleton, the olefinic bond, as a reactive group, could react with sodium hydrogen selenide (NaSeH) via the nucleophilic addition and generate the –SeH. While the –SeH is the typical catalytic center of GPx [28]. Inspired by these, in this work, the OSA starch was firstly employed as the raw material to produce a biodegradable starch-based GPx mimic (Se-starch) via the reaction with NaSeH (Scheme 1). Simultaneously, the synthesis conditions, such as reaction time, reaction temperature, and the component of reactants were optimized to obtain the Se-starch with high catalytic activity. The structural characterizations of the samples were conducted in ^1^H NMR, XPS, SEM-EDS, etc. The catalytic mechanism of Se-starch as a GPx mimic was also studied in vitro. In addition, a MTT assay was used to investigate the cytotoxicity of Se-starch. This study may provide a new method for preparing the Se-functionalized starch with high GPx-like catalytic activity.

## 2. Materials and Methods

### 2.1. Materials

Octenyl succinic anhydride waxy corn starch ester (OSA Starch, food grade) with a degree of substitution (DS) of 2.2%, was purchased from Guangxi State Farms Mingyang Starch Development Co., Ltd., Nanning, China. Chemicals including cumene hydrogen peroxide (CUOOH), hydrogen peroxide (H_2_O_2_), selenium powder, sodium borohydride (NaBH), and 4-nitrothiophenol (NBT) were purchased from J&K Scientific, Ltd., San Jose, CA, USA. Ethanoland sodium hydroxidewere of analytical grade and purchased from Xilong Chemical Co., Ltd., Shantou, China. 3-Carboxy-4 nitrothiophenol (TNB) was synthesized according to the previously reported method [29]. Human hepatocellular carcinoma cell line HepG2 was obtained from Cell Bank of the Type Culture Collection Committee of the Chinese Academy of Sciences.

### 2.2. Preparation of Selenium-Functionalized Waxy Corn Starch

To obtain a NaSeH stock solution, equimolar amounts of Se powder and NaBH_4_ were added to a 100 mL flask, and then an appropriate amount of a mixed solvent of ethanol and H_2_O was slowly added into this flask under N_2_ atmosphere. This flask was held for 2 h at room temperature to get the NaSeH solution. OSA starch (10.0 g) and the mixture of ethanol and H_2_O (100 mL) (the volume ratio of ethanol was 10%, 20%, 30%, 40%, 50%, 60%, 70%, 80%, and 90%, respectively) were placed in a 250 mL flask and vigorously stirred for 30 min. Then, a certain amount of NaSeH solution (the mole ratio of NaSeH to −HC=CH− was 1:1, 1.2:1, 1.5:1, 1.8:1, and 2.2:1, respectively) was slowly added to the above solution in an ice bath, and the pH of the solution was adjusted to the range of 8–10 using NaOH solution (0.1 M). Next, the flask was transferred to a water bath with different temperatures (30, 40, 50, 60, and 70 °C, respectively) for the reaction. After holding for the preset time (between 1 and 8 h), the reaction was terminated by adjusting the solution pH to be about 3 using 0.1% HCl solution. Then, ethanol (about 500 mL) was added into the system over 20 min to precipitate the Se-starch. The starch precipitate was collected by a vacuum filter, and washed successively using 75% ethanol and anhydrous ethanol. Finally, the product was vacuum-dried at 50 °C for 24 h.

### 2.3. Characterizations of Se-Starch

A Bruker Advance III HD 500 MHz NMR spectrometer was used to record the ^1^HNMR spectra of the related samples that were dissolved in D_2_O. Fourier-transform infrared (FT-IR) spectra were collected using a Frontier FT-IR spectrometer (Perkin Elmer) with the attenuated total reflection mode. The scan range of wavenumber was 500–4000 cm^−1^. The scanning electron microscopy (SEM, 6700F, JEOL) and energy-dispersive X-ray spectrometry (EDS) for the Se-starch samples were performed on a 6700F microscope (JEOL). A D8 ADVANCE X-ray diffractometer (Cu-Kα radiation, λ = 0.154 nm; Bruker) was employed to collect the X-ray diffraction (XRD) patterns of the samples over a 2θ range from 5 to 40°, with a tube voltage of 40 kV and a tube current of 40 mA. A Thermo fisher Scientific K-Alpha XPS spectrometer (Mono Al Kα (*h*_v_ = 1486.6 eV), X-ray source of 72 W, wide-scan and high resolution spectra of 100.0 eV and 40 eV, set the C 1s peak to 284.6 eV) was employed to analyze elemental compositions and the selenium form. The Se content of Se-starch was determined using an atomic fluorescence spectrometer (AFS-9530, Beijing Haiguang). The fluorescence spectra of the pyrene-containing samples were recorded on a fluorescence spectrophotometer (Agilent Cary Eclipse) with the excitation wavelength of 335 nm. 

### 2.4. Determination of the Catalytic Activity of Se-Starch

The catalytic activity of sample was determined according to the method reported by Wu and Hilvert [30], using NBT or TNB as an alternative of glutathione (GSH). In a typical test, 700 µL of phosphate buffer (PBS, pH = 7.0), 100 µL of Se-starch dispersion, and 100 µL of NBT or TNB solution (1.5 mM) were added into a quartz cuvette. The mixture in the quartz cuvette was uniformly pre-incubated for 2 min at room temperature. Then, the enzymatic reaction was initiated by adding 100 µL of CUOOH or H_2_O_2_ (2.5 mM) as the substrates. The decrease in the absorption at 410 nm was monitored by an UV spectrophotometer (UV2600, Shimadzu). The catalytic activity (namely the antioxidant performance) of Se-starch was expressed by the initial reaction rate (*v*_0_, μM/min) of the enzymatic reaction, which was calculated by the following equation,

(1) $$v_{0}=\frac{\Delta A}{\varepsilon \times L\times \Delta t}$$

where Δt is the time of the enzymatic reaction from initiation to termination; ΔA is the change of the absorbance during the enzymatic reaction within Δt; ε is the molar extinction coefficient of NBT or TNB (*ε*_(NBT__)_ = 14,600 1/M∙cm, *ε*_(TNB__)_ = 13,600 1/M∙cm, pH = 7.0); L is the optical path length of the quartz cuvette (L = 1 cm). Triplicates experiments were conducted to get the average *v*_0_.

(2) $$v_{0}=\frac{v_{0}\left[S\right]}{\left[S\right]+K_{m}}$$

(3) $$\frac{1}{v_{0}}=\left(\frac{K_{m}}{v_{max}}\right)\frac{1}{\left[S\right]}+\frac{1}{v_{max}}$$

According to the Michaelis and Menten equation (Equation (2)) and double reciprocal plot (Equation (3)), the Michaelis constant (*K*_m_, µM) and the maximum reaction rate (*v*_max_, µM/min) are determined using the Linear-regression curve by plotting 1/*v*_0_ against 1/[S], in which the intercept is 1/*v*_max_ and the intercept is the absolute value of 1/*K*_m_. The [S] is the concentration of substrate [31].

(4) $$K_{\mathrm{cat}}=v_{\max}/\left[E\right]$$

The reaction constant (*K*_cat_, 1/min) was calculated using Equation (4), where the [E] is the concentration of enzyme.

### 2.5. Cell Viability Assays

MTT [3-(4,5-dimethylthiazol-2-yl)-2,5-diphenyltetrazolium bromide] assay was used to measure the cytotoxicity of Se-starch80 toward HepG2 cells according to the previously reported literature [32]. The density of cell was 2 × 10^5^ cells per well. The concentration of the Se-starch80 for incubating the cell varied from 10 to 1000 µg/mL. The optical density was measured using a microplate reader (PerkinElmer Victor X5) at 490 nm. The relative cell viability was depicted as the percentage relative to the optical density derived from the control test.

## 3. Results and Discussion

### 3.1. Preparation and Structural Characterization of Se-Starch 80

In this work, as shown in Scheme 1, thecatalytic center (−SeH) was directly introduced into the OSA starch via the nucleophilic addition between NaSeH and the −HC=CH− in OSA starch. According to the optimization of preparation process for the selenium-functionalized starch (presented later in this work), the Se-starch (Se-starch80) that was produced under the conditions of volume ratio of ethanol 80%, temperature 30 °C, n_(NaSeH__)_:n_(alkene__)_ = 1.5:1 and reaction time 6 h revealed the highest catalytic activity. Such Se-starch with the Se content of 0.033 mg/kg was selected for the characterizations. 

**Figure 1:**
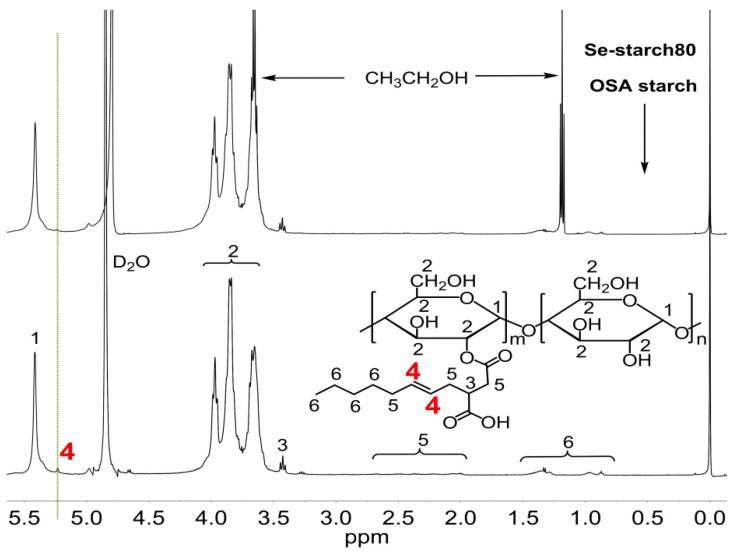
^1^H NMR spectra of OSA starch and Se-starch80 in D_2_O.

**Figure 2:**
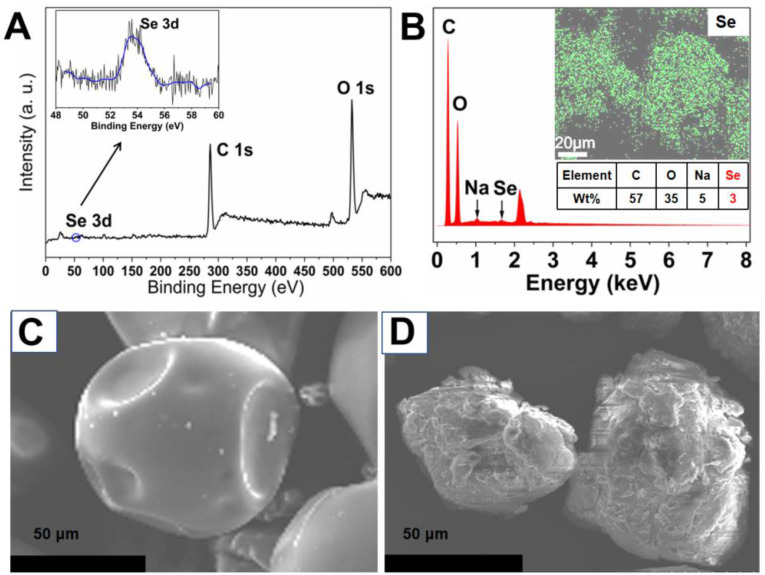
XPS spectrum (**A**), EDS spectrum and Se map of Se-starch80 (**B**); SEM images of OSA starch (**C**) and Se-starch80 (**D**).

In order to determine the selenization reaction mechanism, the ^1^H NMR spectroscopy was used to characterize the changes of OSA starch during the reaction. As shown in Figure 1, for the OSA starch, the ^1^H NMR spectrum showed a proton signal peak at 5.25 ppm, which was ascribed the proton of −HC=CH− in the OSA chains [33]. This peak almost disappeared in the ^1^H NMR spectrum of Se-starch80, suggesting the −HC=CH− participated in the reaction. In this reaction system, NaSeH is a strong nucleophile that can undergo a reaction similar to the −SH and −HC=CH− groups [34]. While the −HC=CH− is the group with the maximum reactivity in the OSA starch toward NaSeH. Therefore, it could be concluded that the nucleophilic addition reaction occurred between NaSeH and −HC=CH−, resulting in the disappearance of the proton signal of −HC=CH−. The selenium in the Se-starch80 was confirmed using XPS, as shown in Figure 2A. The peaks located at 531, 497, 285 and 54 eV were assigned to oxygen, sodium, carbon, and selenium signal, respectively. In the locally magnified image, the Se(3d_3/2, 5/2_) signal peak appeared at about 54 eV, indicating the negative bivalent selenium (Se^2−^) in the Se-starch [35]. Generally, NaSeH can be easily oxidized by O_2_ to form the red nano Se^0^. However, the Se-starch was white, which further proved that the −SeH was covalently bonded to the starch skeleton, rather than being presented as elemental selenium, which is usually gray or red [36]. Furthermore, the EDS results for Se-starch80 showed that the Se was evenly distributed on the surface of the starch particles with a content of 3 wt% (Figure 2B). This content is significantly greater than that of the value determined by AFS (0.033 mg/kg, 3.3 × 10^−6^ wt%). These results suggested that the anchored −SeH was mainly distributed on the surface of starch, which would profit the exposure of the active center for catalytic reaction.

**Figure 3:**
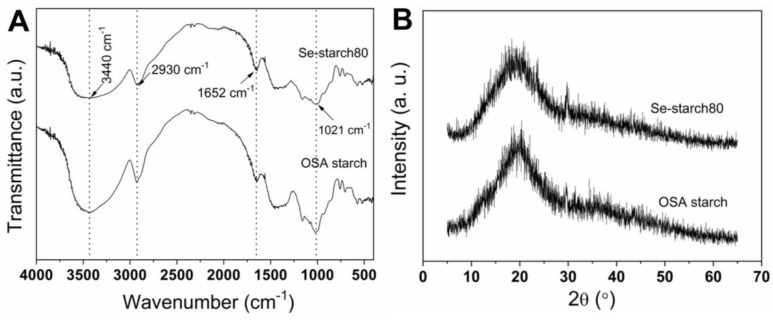
FT-IR spectra (**A**) and XRD patterns (**B**) of OSA starch and Se-starch80.

The OSA starch granule showed a smooth surface (Figure 2C), while a rough surface with grooves was observed in the Se-starch80 (Figure 2D). This morphology change of the starch during the modificationmight be due to the NaSeH, a strong alkali would gelatinize or destruct the starch [37]. The FT-IR spectrum of Se-starch80 presented the characteristic peaks at 3440 cm^−^^1^ (O−H stretching), 2930 cm^−1^ (C−H stretching) [38,39], 1652 cm^−^^1^ (C=O stretching), 1021 cm^−^^1^ (C−O stretching) [40]. These peaks were almost identical to that of OSA starch (Figure 3A). However, the intensity of the O−H stretching (partially relevant to the bound water) for Se-starch80 was lower than that of the OSA starch. One probable explanation is that the outer portion of starch was gelatinized by the NaSeH solution, resulting in a decrease of bound water on the starch surface [41]. In addition, the dehydration of starch by reaction medium with high ethanol content might be the other cause of the decrease of O−H stretching. The structure change for starch during the modification was further studied by XRD (Figure 3B). The XRD pattern for Se-starch80 with a diffraction peak at about 19.4° was similar to that of OSA starch, indicating that the Se-functionalized reaction mainly arose on the surface of starch and did not dramatically damage the internal skeleton of starch [41]. 

### 3.2. Optimization of the Preparation Process of Se-Starch

**Figure 4:**
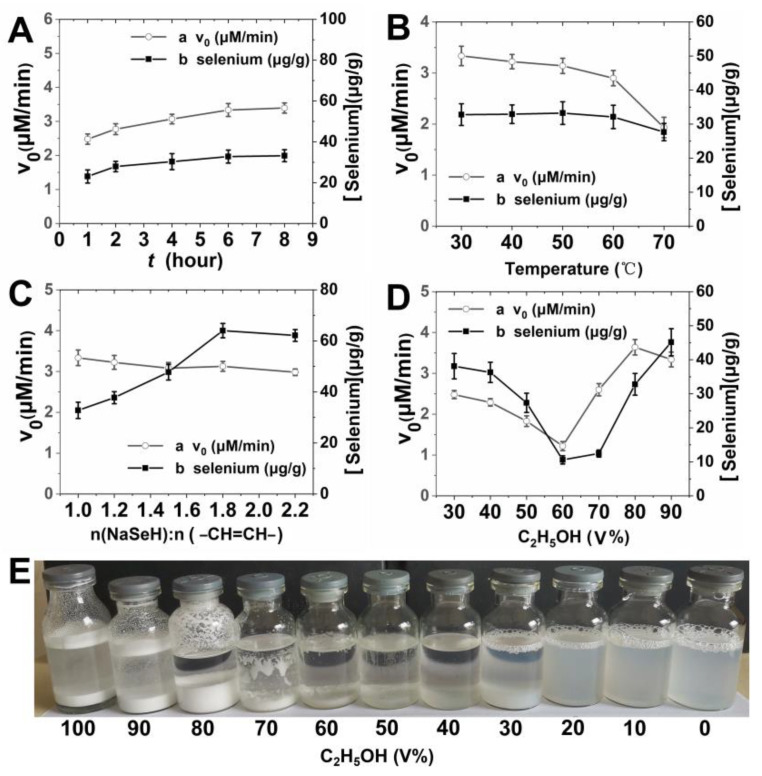
The *υ*_0_ and the selenium content as the function of reaction time (**A**), reaction temperature (**B**), the mole ratio of NaSeH and −HC=CH− (**C**), and volume ratio of ethanol (**D**); the digital photographs of the reaction system with different volume ratio of ethanol (**E**).

Similar to natural enzymes, the catalytic activity of GPx mimic may be greatly affected by structural changes in their skeleton, catalytic center, and peripheral recognition [12,24]. For the Se-starch, the starch provided a degradable framework with −SeH as the catalytic center, and formed a coarse surface with grooves, which was similar to the natural GPx. In addition, the OSA molecular chains offered the Se-starch surface with hydrophobic microenvironment, which could also influence the catalytic activity. These factors would be affected by the reaction condition. Therefore, the effect of the reaction conditions including reaction time, reaction temperature, n_(NaSeH)_:n_(-HC=CH-)_, and volume ratio of ethanol (V%) on the Se content and catalytic activity (*υ*_0_, μM/min) were investigated. The NBT and CUOOH were used as the substrates to test the *υ*_0_. Figure 4A shows the effect of reaction time on the Se content and *υ*_0_ under the conditions of reaction temperature 40 °C, n_(NaSeH)_:n_(−HC=CH−)_ = 1:1 and volume ratio of ethanol 30%. The *υ*_0_, similar to the Se content, increased with the prolongation of reaction time in the initial stage of 6 h. Due to the equilibrium of reaction, the further increase of reaction time did not promote the *υ*_0_ and the Se content significantly. Therefore, the reaction time of 6 h was selected for preparing Se-starch.

The effect of reaction temperature on the Se content and *υ*_0_ under the conditions of reaction time 6 h, n_(NaSeH)_:n_(−HC=CH−)_ = 1:1, and volume ratio of ethanol 30% was conducted by altering the temperature from 30 to 70 °C (Figure 4B). The Se content almost did not changed at the temperature range from 30 to 60 °C, while dropped remarkably at 70 °C. A possible explanation is that the reverse reaction of the nucleophilic addition reaction was dominant at high temperature. For the *υ*_0_, it decreased slightly as the temperature increased from 30 to 60 °C and declined significantly at 70 °C. The reduction of *υ*_0_ would attribute to the decrease of Se. The partial gelatinization of the starch at high temperature would cover the Se active center, which might be the other reason for the decrease of *υ*_0_. Therefore, the nucleophilic addition reaction between NaSeH and OSA starch was performed at 30 °C. The influence of the NaSeH content on the reaction was studied by changing the n_(NaSeH)_:n_(−HC=CH−)_ from 1.0 to 2.2 at the conditions of reaction time 6 h, reaction temperature 30 °C, and volume ratio of ethanol 30%. As shown in Figure 4C, the Se content increased with the increase of the molar ratio of NaSeH and −HC=CH−, whereas the *υ*_0_ did not change significantly as the molar ratio of NaSeH and −HC=CH− increased. This result might be ascribed to the partial gelatinization of starch by alkaline NaSeH, which coated the Se active center and hindered its catalysis. Although the high content of NaSeH could promote the Se content of Se-starch, the excessive NaSeH resulted in a troublesome purification of the product. Therefore, the molar ratio of NaSeH and −HC=CH− was set to be 1.5 for the further experiments. 

Figure 4D reveals the effect of volume ratio of ethanol (V%) on the Se content and *υ*_0_ under the conditions of reaction time 6 h, reaction temperature 30 °C, and n_(NaSeH)_:n_(−HC=CH−)_ = 1.5:1. At the low volume ratio of ethanol (such as V% = 0, 10, and 20%), the OSA starch was completely emulsified and dispersed in the solution, resulting in a difficulty for separating the products from the solution (Figure 4E). The increase of the V% from 30% to 60% caused the aggregation of the soluble OSA starch, forming a bulk precipitation with high adhesion. This cohesive precipitation with limited reactive site to be exposed would restrict the nucleophilic addition reaction, resulting in a low Se content and catalytic activity. As the further increase of the V%, the OSA starch particles could be dispersed freely in the reaction system, which benefited the heterogeneous reaction between NaSeH and OSA starch and consequently caused the increase of the Se content as well as the *υ*_0_. The decline of the *υ*_0_ at V% = 90% might be due to the dehydration of starch by ethanol, which could compact the starch to decrease the exposure of catalytic center. In addition, the change of the surface property of starch by dehydration might be the other reason of the decline of the *υ*_0_ at V% = 90%. Therefore, the optimized volume ratio of ethanol for the reaction system was 80%. Taken together, the optimized reaction conditions for preparing Se-starch were as follow: reaction time 6 h, temperature 30 °C, molar ratio of NaSeH to −HC=CH− 1.5 and volume ratio of ethanol 80%, in which the targeted Se-starch (Se-starch80) revealed a high catalytic activity (*υ*_0_ = 3.64 μM/min). This value is 1.5 × 10^5^ times higher than the catalytic activity of a typical small-molecule GPx mimic (PhSeSePh) at the reaction system of NBT and CUOOH.

### 3.3. Catalytic Mechanism and Catalytic behavior of Se-Starch80

**Scheme 2:**
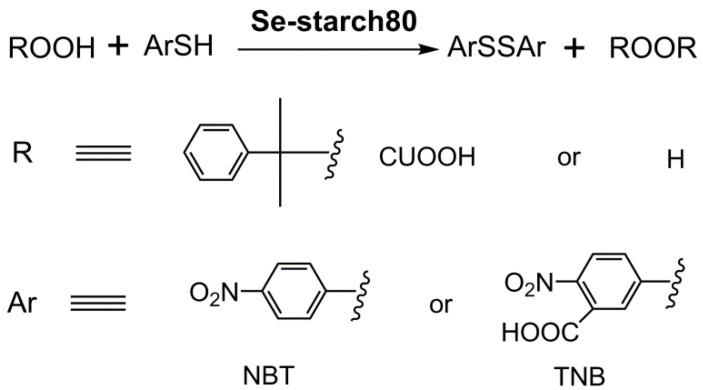
The reaction between thiophenol substrates and hydroperoxide substrates under catalysis of Se-starch80.

**Figure 5:**
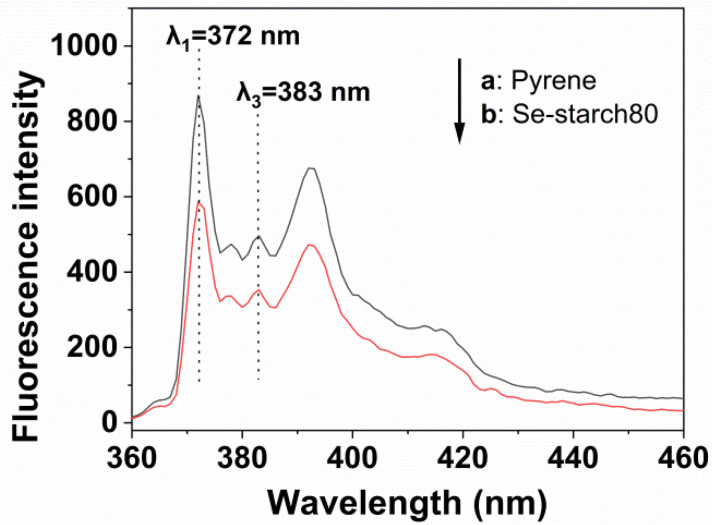
Fluorescence spectra of pyrene solution and the pyrene/Se-starch80 solution, where the concentration of pyrene was 1.0 × 10^−6^ M.

In general, the combinations with different substrates were used to analyze the effect of the hydrophobic interaction and the substrate recognition sites between substrates and the catalyst [42,43]. As the typical substrate, 4-nitrothiophenol (NBT, a hydrophobic substrate) and 3-carboxy-4-nitrothiophenol (TNB, a hydrophilic substrate) were used as the reductive thiophenol substrates, while H_2_O_2_ and CUOOH were used as the hydroperoxide substrates. H_2_O_2_ is more hydrophilic than the CUOOH for the absence of the p-cumyl group in H_2_O_2_. These substrate combinations were employed to test the catalytic activity of Se-starch80 (Scheme 2), in which the concentrations of thiophenol substrate and hydroperoxide substrate were 150 µM and 250 µM, respectively. As shown in Table 1, Se-starch80 revealed different *υ*_0_ at the reaction systems with various substrates. In the system containing both hydrophobic substrates (NBT + CUOOH), Se-starch80 exhibited the highestυ_0_, while it showed the minimumυ_0_ in the system of TNB and H_2_O_2_, two hydrophilic substrates. These results suggested that the hydrophobic interaction played an important role in the catalysis. The hydrophobic microenvironments in the Se-starch80 were testified by the pyrene fluorescence probe experiment [44] (Figure 5). The pyrene solution (C = 1.0 × 10^−6^ M) revealed a fluorescence intensity ratio (I_1_/I_3_, the indicator of hydrophobicity) of 1.81 at peak 1 (372 nm) and peak 3 (383 nm). However, the solution containing pyrene (concentration = 1.0 × 10^−6^ M) and Se-starch80 with a lower I_1_/I_3_ of 1.66, indicating the formation of hydrophobic microenvironments in the Se-starch80 for gathering pyrene molecule [45]. The OSA starch, without −SeH, did not present any catalytic activity in the system of NBT + CUOOH, suggesting the key function of Se in the catalysis. In the system of TNB + CUOOH, the *υ*_0_ of Se-starch80 was comparable to that of Micellar Catalyst (Table 1), a previously reported GPx mimic with high catalytic activity [46].

**Figure 6:**
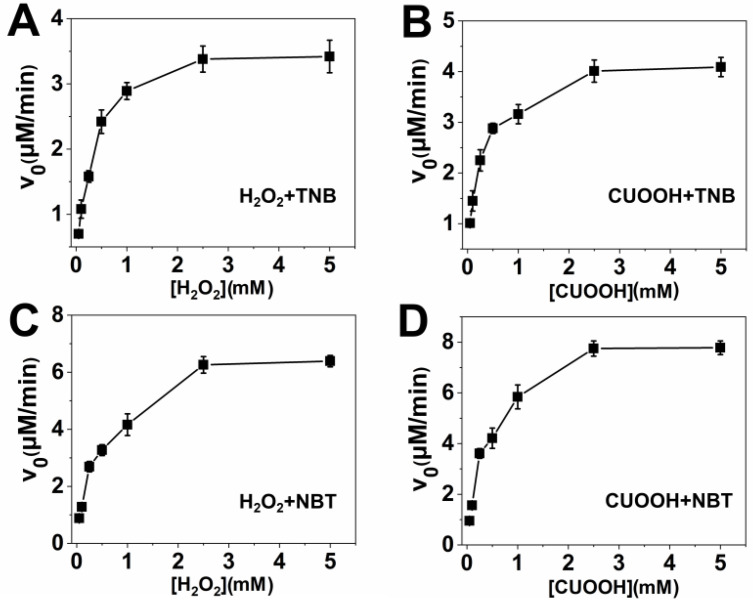
The initial rates as the function of concentration of hydroperoxide substrates at various reaction systems, H_2_O_2_ + TNB (**A**), CUOOH + TNB (**B**), H_2_O_2_ + NBT (**C**), and CUOOH + NBT (**D**).

The catalytic behavior of Se-starch80 was further investigated by changing the concentration of hydroperoxide substrates at the system containing the thiophenol substrates (0.15 mM). As shown in Figure 6, for all substrate combinations, the catalytic activity of Se-starch80 increased with the rise of the concentration of hydroperoxide substrates and then arrived at the equilibrium, suggesting a saturation kinetic catalytic behavior. This catalytic behavior was similar to a typical catalytic behavior of enzyme. Based on the profiles of *υ*_0_ versus the concentrations of hydroperoxide substrates, some catalytic kinetic parameters such as the maximum reaction rate (*v*_max_, µM/min), the reaction constant (*K*_cat_, 1/min), Michaelis‒Menten constant (*K*_m_, µM), and the catalytic efficiency (*K*_cat_/*K*_m_, 1/M∙min) were calculated and listed in Table 2. The *K*_m_, an index of the affinity between catalyst and substrates, designates the concentration of substrate at *v* = 0.5 *v*_max_, where *v* is the reaction rate. A lower *K*_m_ indicates a stronger affinity between catalyst and substrates. Taking the reaction system of CUOOH + TNB and the system of CUOOH + NBT as the comparison, the *K*_m_ of Se-starch80 at the system of CUOOH + TNB is inferior to the *K*_m_ derived from the system of CUOOH + NBT, indicating that Se-starch80 revealed a higher affinity to NBT than TNB. The higher affinity for TNB could be due to the bearing of –COOH, which could interact with the hydroxyl of starch via hydrogen bond. However, the catalytic activity (*υ*_0_) and the catalytic kinetic parameters including *υ*_max_, *K*_cat_, and *K*_cat_/*K*_m_ of Se-starch80 in the system of CUOOH + TNB were smaller than that in the system of CUOOH + NBT. A possible explanation was that the strong affinity of Se-starch80 toward TNB resulted in a poor dissociation of the substrate from catalyst after catalytic reaction. The active centers in the catalyst were occupied by the bonded substrates, which hindered the subsequent reaction and consequently decreased the catalytic activity of Se-starch80.

### 3.4. Cytotoxicity of Se-Starch80

**Figure 7:**
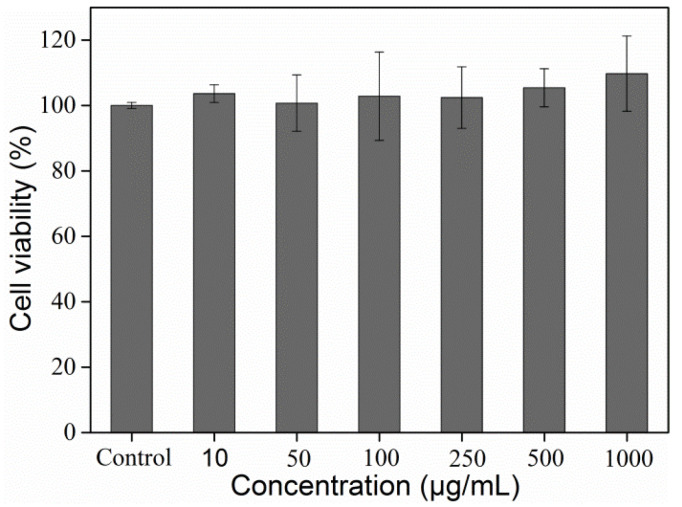
The cell viability as the function of the concentration of Se-starch80.

The exposure of Se-starch80 to the proliferation of HepG2 cancer cells was conducted to test the cells growth in a concentration-dependent manner (Figure 7). For all Se-starch80 concentrations in the media (10–1000 g/mL), the HepG2 cell viability was marginally greater than 100%, suggesting the facilitation of cell proliferation by Se-starch80. This result might be attributed to the antioxidative activity of Se-starch80 as a GPx mimic. Overall, the Se-starch80, without any cytotoxicity, promoted the cell growth, suggesting that the biodegradable selenium-functionalized corn starch may be a good candidate for antioxidant enzyme mimic [23,47].

## 4. Conclusions

In this paper, a new strategy for producing the Se-functionalized starch with GPx-like catalytic activity was developed by the nucleophilic addition between NaSeH and OSA waxy corn starch. The covalently linked −SeH on the starch surface provided it with catalytic centers, while the OSA chains supplied the hydrophobic microenvironments for gathering substrates. The optimal technological conditions for preparing Se-functionalized starch were that reaction time 6 h, temperature 30 °C, molar ratio of NaSeH to −HC=CH− 1.5 and volume ratio of ethanol 80%. Such Se-functionalized starch with a Se content of 0.033 mg/kg revealed a high capacity for catalyzing the reaction between CUOOH and NBT (*v*_0_ = 3.64 μM/min). The high catalytic activity of Se-functionalized starch was attributed to the intensive distribution of catalytic center on the surface of starch and the hydrophobic microenvironments. The Se-functionalized starch did not require any bioconversion to perform the GPx-like function and showed a typical saturated kinetic catalytic behavior. In addition, the biodegradable starch skeleton did not change significantly during the modification and such Se-functionalized starch had no toxicity to cells. Therefore, a non-toxic Se-starch was prepared by a chemical modified method, which is expected to become a novel supplement with GPx-like activity for solving the nutrient deficiency of selenium. This work may provide a potential for the preparation of Se-functionalized starch.

## Figures and Tables

**Table 1:** The initial rates (*v*_0_) for the reduction of ROOH (250 µM) by ArSH (150 µM) in the presence of Se-starch80 at 25 °C and pH 7.0 (50 mM PBS).

Catalyst	ArSH	ROOH	*υ*_0_ (μM/min) ^a^
PhSeSePh	NBT	CUOOH	2.38 × 10^−5^
Micellar Catalyst	TNB	CUOOH	2.25 ± 0.24 [41]
OSA starch	NBT	CUOOH	ND ^b^
Se-starch80	TNB	CUOOH	2.11 ± 0.17
Se-starch80	TNB	H_2_O_2_	1.58 ± 0.09
Se-starch80	NBT	CUOOH	3.64 ± 0.19
Se-starch80	NBT	H_2_O_2_	2.69 ± 0.18

^a^ Main±SD, each sample was measured for three times, and the *v*_0_ was calculated on the basis of 1.0 μM selenium monomer; ^b^ ND, not detected.

**Table 2:** The catalytic saturation kinetic constants of Se-starch80 in different reaction systems.

Substrates	*v*_max_	*K* *_cat_*	*K* *_m_*	*K_cat_/ K_m_*
	(µM/min)	(1/min)	µM	(×10^5^ 1/M∙min)
CUOOH + NBT	7.94	15.88	750.79	2.11
H_2_O_2_ + NBT	5.55	11.10	558.89	1.99
CUOOH + TNB	3.75	7.50	281.65	2.66
H_2_O_2_ + TNB	3.24	6.48	373.46	1.73

## Data Availability

The data presented in this study are available on request from the corresponding author.
